# Clinical, histopathological and immunohistochemical analysis of vulvar squamous cell carcinoma

**DOI:** 10.61622/rbgo/2024rbgo91

**Published:** 2024-12-04

**Authors:** Lorenza Bridi Todeschini, Rita de Cássia Sant’Anna Alves, Adriana Vial Roehe

**Affiliations:** 1 Universidade Federal de Ciências da Saúde de Porto Alegre Porto Alegre RS Brazil Universidade Federal de Ciências da Saúde de Porto Alegre, Porto Alegre, RS, Brazil.

**Keywords:** Vulvar squamous cell carcinoma, Immunohistochemistry, p16, p53, PDL1

## Abstract

**Objective:**

The average age of patients with vulvar squamous cell carcinoma (SCC) has been reported to have declined. Human papilloma virus (HPV)-related lesions have been shown to be associated with the expression of the immunohistochemical (IHC) marker p16. Non-HPV-related tumors have been characterized by p53 abnormal expression and PDL1 expression. We aimed to evaluate the correlation between these markers and vulvar SCC and to relate it to the clinical and pathological characteristics.

**Methods:**

Histopathologic assessments and IHC analyses of p16, p53, and PDL1 were performed in 41 samples of vulvar SCC collected between 2016 and 2021. The data were correlated with clinical and pathological characteristics of the patients.

**Results:**

The mean age of the patients was 72.1 years. Positive p16 and PDL1 staining was detected in 24.4% and 17.1% of the samples, respectively. p53 expression was negative in 19.5% of the samples, whereas it was overexpressed in 24.4%. p16-positive tumors showed a smaller depth of invasion (DOI) (p = 0.014), while tumors with p53 abnormal expression showed greater DOI (p = 0.041). PDL1 expression was correlated with increased number of inflammatory cells (p = 0.055). In addition, lesions with lymphovascular space invasion were p16-negative.

**Conclusion:**

In our sample, regarding to the SCC incidence the patients’ mean age did not change. The expression of p16 was inversely correlated with p53 results. Tumors with p53 abnormal expression and absence of p16 showed a greater DOI. Our data suggest an association between PDL1 expression and increased inflammatory infiltrates in vulvar SCC.

## Introduction

Vulvar cancer is a rare entity, corresponding to approximately four to five percent of gynecological cancers and less than one percent of the total of cancers in women.^(1–3)^ In the last four decades, an increase in the incidence of vulvar squamous cell carcinoma (SCC) has been reported in younger women. Kang et al.^(4)^ have evaluated data from 13 high-income countries for 20 years and observed a 14% increase in the incidence of vulvar cancer. The overall rise was greater in patients aged < 60 years.^(4)^ The 2021 edition of the International Federation of Gynecology and Obstetrics (FIGO) Cancer Report associated the decline in the average age of SCC incidence with an increase in human papilloma virus (HPV) infections, a risk factor for vulvar cancer.^(1–3,5)^

Recent studies have shown that HPV-related vulvar SCC has a better prognosis compared to HPV-unrelated cases.^(6–9)^ SCCs HPV-unrelated tend to have more aggressive behavior and bad outcome.^(10)^ However, morphology alone does not allow an accurate differentiation between the two subtypes. So, the expression of the IHC marker p16 has been used to identify HPV-related cases.^(9,11,12)^

P53 abnormal expression is present in approximately two-thirds of vulvar cancers HPV-unrelated.^(11,13,14)^ The mutation pattern of p53 denotes the importance of oxidative stress in the etiopathology of vulvar carcinogenesis, for example, in cases related to lichen sclerosus.^(15)^

Programmed Death Ligand 1 (PDL1) helps the tumor cells to escape the immune system detection by binding to the Programmed Cell Death 1 (PD1), a cell death receptor expressed on T lymphocytes. PD1/PDL1 negatively regulates the proliferation and function of T lymphocytes, allowing the tumor to remain undetected by the immune system.^(16,17)^ In vulvar SCC, PDL1 expression occurs more frequently in HPV-negative cases.^(16)^ Patients with vulvar carcinomas with high PDL1 expression have a higher risk of disease recurrence and mortality.^(18)^ Blocking the PD1/PDL1 interaction offers an alternative immunotherapeutic strategy, and patients with accentuated inflammatory infiltrate in the tumor appear to respond better to this treatment.^(19–22)^

Studies addressing the etiology and age-associated incidence pattern of vulvar cancer in the Brazil are limited. Therefore, the evaluation of the patients’ profile may help to establish prognostic and predictive criteria for SCC patients. In this study, we aimed to evaluate the clinical and pathological aspects of vulvar SCC to identify possible correlations with potential clinical implications.

## Methods

A cross-sectional study was conducted using a database of videovulvoscopic images (Defferrari SDI^®^, Novo Hamburgo, BR) and histological samples from biopsies of lesions diagnosed as vulvar SCC from 41 patients. Videovulvoscopy and histological analyses were performed between January 2016 and December 2021 at the Citoclin Laboratory (Porto Alegre, BR). A total of 28 samples were collected in the laboratory and 13 samples were collected at state hospitals, and later sent to the Citoclin Laboratory for histopathological analysis. Patients whose biopsies were collected at other institutions and sent for laboratory analysis did not have a vulvoscopic image for correlation.

Were included in the study patients with age ≥ 18 years old with anatomopathological diagnosis of SCC.

Patients whose biopsy material was scarce were excluded from the study.

Videovulvoscopy was performed with image magnification using a videocolposcope (Elemed^®^ Porto Alegre, BR) after application of five percent acetic acid. The images were analyzed by two colposcopists qualified for colposcopy and analysis of lower genital tract pathology. The dermatological characteristics of the lesion, anatomical location, number of lesions, approximate size in centimeters (cm), and diagnostic impression of the lesion were evaluated.

Hematoxylin and eosin (HE) stained slides of the samples were reviewed by two pathologists. Histopathological analysis confirmed the histological type, deep tumoral invasion (DOI) in the biopsy specimen, presence of ulceration and lymphovascular invasion. Inflammatory infiltrate was classified as mild, moderate, or severe. All the analyses were conducted in the Pathology Laboratory at UFCSPA.

IHC analysis of p16 was carried out on the automated Roche Ventana platform with the ready-to-use mouse monoclonal anti-p16 antibody clone E6H4 (Roche^®^ Arizona, USA). P16 positive status was determined based on brown staining of at least 70% of the tumor, including the cell nucleus, according to the criteria of the American College of Pathology.^(23)^

Immunostaining for p53 was performed manually using mouse monoclonal anti-p53 antibody (catalog number m7001, dilution 1:1000, Dako^®^ Carpinteria, USA). P53 expression was considered to be abnormal when there was a complete absence of staining or when ≥50% of the tumor cells showed nuclear staining.^(24)^

PDL1 immunostaining was performed manually using an anti-PDL1 antibody (catalog number ACR3171C, dilution 1:150, Biocare® Concord, USA). Positive expression was defined as any extent of membranous staining in ≥1% of the tumor cells.^(18)^

Categorical variables were presented as absolute frequencies and percentages. Quantitative variables were presented as mean and standard deviation. Normality was assessed by Shapiro-Wilk test. The Mann-Whitney and Kruskal-Wallis tests were used to compare the DOI and lesion size with IHC markers. Student's t-test and ANOVA were used for age analysis. Other associations were verified using Fisher's Exact and Chi-Square tests. Statistical significance was set at P < 0.05. Analyses were performed using SPSS statistical software (IBM SPSS Statistics for Windows, Version 25.0. Armonk, NY: IBM Corp., USA).

The project was submitted and approved by the Research Ethics Committee of the Federal University of Health Sciences of Porto Alegre (CEP - UFCSPA - Protocol number: 3.084.049). The Ethics Committee waived the need of informed consent.

## Results

A total of 41 cases of vulvar SCC were analyzed, of which 28 had videovulvoscopic images available for correlation. The average age of the patients was 72.1 years old (range: 45–90 years old). Of the 28 cases that had videovulvoscopic images available, 20 (71.4%) were Caucasian and eight (28.6%) were black. In these 28 cases, most lesions were located in left labia majora (n=9, 29%), followed by right labia minora (n=7, 22.6%), and vaginal introitus (n=7, 22.6%). Most patients had only one lesion (n=25, 89.3%). Three (20.7%) patients had two concomitant SCC lesions. The most frequent lesion type was vegetative/exophytic (51.6%), followed by friable lesion 11 (35.5%). The mean size of the vulvoscopic lesions were 2.5 cm (range: 0.4–5 cm). The diagnostic impression of videovulvoscopy examiners was accurate in 18 (69.2%) of the cases (Tables 1 and 2).

**Table 1:** Pathological and immunohistochemical characteristics of the cases

Variables		n(%)	Mean	SD*
Age (n = 41)			72.1	12.5
Type of SCC (n = 41)	Keratinized	31(75.6)		
	Keratinized and verrucous	3(7.3)		
	"Warty"	1(2.4)		
	Keratinized and ulcerated	3(7.3)		
	Keratinized and basaloid	3(7.3)		
Ulceration (n = 41)	Yes	20(48.8)		
	No	21(51.2)		
Inflammatory infiltrated (n = 41)	Mild	19(46.3)		
	Moderated	19(46.3)		
	Accentuated	3(7.3)**		
Lymphovascular space invasion (n = 41)	Yes	2(4.9)		
	No	39(95.1)		
Depth tumor invasion (mm) (n = 41)			1.6 (0-5-6.2)	
p16 (n = 41)	Positive	10(24.4)		
	Negative	31(75.6)		
p53 (n = 41)	0%	8(19.5)		
	1%-9%	3(7.3)		
	10%-24%	13(31.7)		
	25%-49%	7(17.1)		
	>=50%	10(24.4)		
PDL1 (n=41)	Positive	7(17.1)		
	Negative	34(82.9)		

* SD- Standard deviation;

** Two of those were PDL1 positive (p = 0.055)

**Table 2:** Vulvoscopy characteristics

		n(%)	Mean	SD*
Skin color (n = 28)	White	20(71.4)		
	Black	8(28.6)		
Diagnostic impression on vulvoscopy (n = 26)**	SCC	18(69.2)		
	HPV	1(3.8)		
	VIN	6(23.1)		
	Other	1(3.8)		
Vulvoscopic characteristics of the lesions (n = 31)***				
Size of the lesion (cm)			2.5	1.4
Location	Right labia minora	7(22.6)		
	Left labia minora	4(12.9)		
	Right labia majora	4(12.9)		
	Left labia majora	9(29.0)		
	Vaginal Introitus	7(22.6)		
	Perianal	1(3.2)		
	Perineum	3(9.7)		
	Clitoris	3(9.7)		
Type of vulvoscopic lesion	Ulceration	8(25.8)		
	Keratosis	6(19.4)		
	Vegetative/exophytic	16(51.6)		
	Pigmented	2(6.5)		
	Papulous	1(3.2)		
	Lichenoid	4(12.9)		
	Friable	11(35.5)		
	Grainy	2(6.5)		
	Achromia	3(9.7)		
	Polypoid	1(3.2)		
	Warty	1(3.2)		
	Acetowhite epithelium	3(9.7)		
	Signs of necrosis	3(9.7)		
	Atypical vessels	4(12.9)		
	Nodular	1(3.2)		
	Erosion	1(3.2)		
Number of lesions in each patient in vulvoscopy (n = 28)	One	25(89.3)		
	Two	3(10.7)		

* SD- Standard deviation,

** 2 of the cases did not have diagnostic impression,

*** n = 31 because three patients had two lesions each, plus 25 patients with one lesion each

Regarding to the anatomopathological aspects, keratinized SCC was the most frequent histological type 31 (75.6%). Ulceration was present in approximately 20 (48.8%) of the cases. Inflammatory cell infiltrate was mild in 19 (46.3%), moderate in 19 (46.3%), and accentuated in three (7.3%) cases. The mean DOI was of 1.6 mm (range: 0.5–6.2 mm). Lymphovascular space invasion was observed in two (5%) cases. Considering the IHC markers, p16 was positive in 10 (24.4%) and PDL1 was positive in seven (17.1%) patients. P53 expression was abnormal in 18 (43.9%) cases: absent in eight (19.5%) and overexpressed in 10 (24.4%) of the cases. The expression of p16 and p53 were inversely correlated: p16-positive cases had a normal expression of p53; p16-negative cases, presented p53 abnormal expression (p = 0.025). There was no significant correlation between PDL1 and p16 expression, nor PDL1 and p53 expression (Table 3).

**Table 3:** Correlation between expression of immunohistochemical markers p16, p53, and PDL1, and deep tumor invasion in biopsy samples of vulvar SCC (n = 41)*

Mean		Deep tumor invasion in the biopsy sample (mm)		
		SD**	p-value	
p16	Positive	1.1	0.4	0.014
	Negative	1.8	1.0	
p53	1%-49%	1.4	0.6	0.041
	0% or >=50%	1.9	1.2	
PDL1	Positive	2.2	1.9	0.578
	Negative	1.5	0.6	

* Student's t-test and analysis of variance were used for age × markers. The Mann-Whitney's and Kruskal-Wallis tests were used to determine the correlation between depth of tumoral invasion in the biopsy sample × markers, as normality was not observed in the Shapiro-Wilk test;

** Standard deviation

The average DOI was 1.1mm in p16-positive cases *versus* 1.8mm in p16-negative ones (p = 0.014). In cases with p53 abnormal expression, the mean DOI was 1.9mm, *versus* 1.4mm in the group with normal p53 expression (p= 0.041). (Table 3). Regarding to the inflammatory infiltrate, the majority of those with accentuated inflammatory cells (two of three cases) were PDL1-positive (p = 0.055). Lymphovascular space invasion was observed only in two cases: both were p16- negative, and one was PDL1-positive with abnormal p53 expression.

## Discussion

This study was designed to evaluate the clinical, histological, and immunohistochemical profiles of patients with vulvar SCC in Southern Brazil over a six-year period. The profile of our patients was mostly composed by older patients (mean age of 72.1 years old), with p53 abnormal expression and a mean DOI of 1.6mm. The lymphovascular space invasion was not a frequent event.

In contrast to previous reports, we did not find a decline in the mean age of patients with vulvar SCC.^(1–5)^ Four patients were under 60 years of age: three of them aged over 50 years, and only one was 45 years old patient with a previous history of vaginal high grade squamous intraepithelial lesion (HSIL) caused by HPV.

In terms of location, most of the lesions were in the labia majora and minora, which is consistent with a previous report.^(3)^

The histopathological analysis showed no correlation with IHC markers, reassuring that the morphology is not pathognomonic of vulvar SCC etiology.^(12)^ In our samples, 25.8% of keratinized SCC were p16-positive, suggesting an HPV correlation, despite the anatomopathological aspect.

In our study the frequency of cases with p53 abnormal expression (44%) was higher than p16 positivity (24%). It suggests, in contrast to previous reports, that the most common etiology of vulvar SCC in our sample was not HPV-related.^(1–3)^ We also observed an inverse correlation between p53 and p16 expressions, demonstrating that, although the HPV carcinogenic pathway is known to affect p53 expression, this is not very frequent in vulvar SCC.^(13)^ Considering the DOI in the biopsy specimens, we found interesting data. The average DOI was 1.1 mm in p16-positive cases and 1.9 mm in cases with p53 abnormal expression, inferring that the DOI tend to be greater in vulvar SCCs not HPV-related. It is important to point out that evaluation of the DOI can be challenging in biopsy specimens, especially when it is less than 1 mm, because of the possibility of underestimating the real DOI.^(25,26)^However, in cases where the DOI is >1 mm, it would be helpful for accurately approaching the inguinal lymph nodes. In our sample, the mean DOI was 1.6 mm, which suggests that in most cases the patients’ inguinal lymph node compartment would need to be evaluated.

We found no association between lymphovascular space invasion and p16 expression. However, it was observed in only two cases in our cohort. Both cases were p16-negative, one of them was PDL1-positive and another one had p53 abnormal expression. It should be considered that the small number of cases evaluated could affect these data correlation.

Previous studies suggest that the presence of inflammatory cells infiltrates may be related with a better prognosis, since it is a sign of immune response.^(22,27)^ In our sample, there was an association of PDL1 expression and more accentuated inflammatory cells infiltrate. This may be predictive of better response to immunotherapies targeted at blocking the PD1/PDL1 interaction.^(21)^

The main limitations of our study are the sample size and the fact that the histological analysis was performed in punch biopsies specimens, without correlation with the final anatomopathological analysis. However, we should ponder the scarcity of vulvar SCC *per se* and of the studies about it. Despite these limitations, we believe that our study brings valuable information to the clinical, histological, and immunohistochemical profile of patients with vulvar SCC in Southern Brazil.

## Conclusion

Our study results did not show a reduction in the mean age of vulvar SCC patients, considering the classical age of the onset of the malignancy (usually ≥60 years old). Most vulvar SCC cases in the study were not HPV-related. These two findings suggest that vulvar SCC etiology pattern did not change in our sample. We identified an inverse association between p53 abnormal expression and p16 expression, and a direct relation between a higher DOI and p53 abnormal expression. It was observed an association of PDL1 expression and the presence of accentuated inflammatory cells infiltrate. Further studies with larger sample size and follow up of the patients would help to elucidate the clinic and pathological profile for vulvar SCC and their prognostic impact in the future.
